# Lipoprotein Lipase Regulates Microglial Lipid Droplet Accumulation

**DOI:** 10.3390/cells10020198

**Published:** 2021-01-20

**Authors:** Bailey A. Loving, Maoping Tang, Mikaela C. Neal, Sachi Gorkhali, Robert Murphy, Robert H. Eckel, Kimberley D. Bruce

**Affiliations:** 1 Department of Radiation Oncology, Oakland University William Beaumont School of Medicine, Royal Oak, MI 48309, USA; bailey.loving@beaumont.org; 2 Division of Endocrinology, Metabolism and Diabetes, University of Colorado, Anschutz Medical Campus, Aurora, CO 80045, USA; maoping.tang@cuanschutz.edu (M.T.); mikaela.neal@cuanschutz.edu (M.C.N.); sachigorkhali@gmail.com (S.G.); robert.eckel@cuanschutz.edu (R.H.E.); 3 Department of Pharmacology, University of Colorado Denver, Anschutz Medical Campus, Aurora, CO 80045, USA; robert.murphy@cuanschutz.edu

**Keywords:** lipoprotein lipase, PPAR agonists, microglia, neurodegenerative disease, lipid droplet

## Abstract

Microglia become increasingly dysfunctional with aging and contribute to the onset of neurodegenerative disease (NDs) through defective phagocytosis, attenuated cholesterol efflux, and excessive secretion of pro-inflammatory cytokines. Dysfunctional microglia also accumulate lipid droplets (LDs); however, the mechanism underlying increased LD load is unknown. We have previously shown that microglia lacking lipoprotein lipase (LPL KD) are polarized to a pro-inflammatory state and have impaired lipid uptake and reduced fatty acid oxidation (FAO). Here, we also show that LPL KD microglia show excessive accumulation of LD-like structures. Moreover, LPL KD microglia display a pro-inflammatory lipidomic profile, increased cholesterol ester (CE) content, and reduced cholesterol efflux at baseline. We also show reduced expression of genes within the canonical cholesterol efflux pathway. Importantly, PPAR agonists (rosiglitazone and bezafibrate) rescued the LD-associated phenotype in LPL KD microglia. These data suggest that microglial-LPL is associated with lipid uptake, which may drive PPAR signaling and cholesterol efflux to prevent inflammatory lipid distribution and LD accumulation. Moreover, PPAR agonists can reverse LD accumulation, and therefore may be beneficial in aging and in the treatment of NDs.

## 1. Introduction

Microglia are brain-resident macrophages that constitute the largest population of immune cells in the adult central nervous system (CNS). They play a major role in maintaining homeostasis within the brain and can initiate, modulate, and resolve inflammation in disease [1,2]. Due to their heterogeneity and pleiotropic nature, considerable efforts have been made to understand the phenotypic characteristics of microglia that may either prevent or predispose disease. Recently, several single-cell RNA sequencing (scRNAseq) studies have defined the transcriptomic identities of microglial subpopulations with temporal [3,4,5], regional [3,4,5], and disease state specificity [4,6,7]. These analyses have shown that microglial lipid metabolism is tightly regulated during development, damage, and disease [8]. For example, disease-associated microglia (DAM) express a distinct set of genes associated with lipid and lipoprotein metabolism (e.g., Apolipoprotein E [ApoE], Lipoprotein Lipase [LPL], and Triggering Receptor Expressed On Myeloid Cells 2 [TREM2]) [7]. DAMs are thought to be phagocytic and protective, since microglia from Alzheimer’s disease (AD) patients and 5XFAD mice express LPL and internalize amyloid-beta (Aβ) [7]. A similar signature is observed in the proliferative-region-associated-microglia (PAMs) of the early post-natal brain [3], microglia of the demyelinating brain [4], and microglial subpopulations observed in the later stages of DNA damage and neurodegeneration [7]. Nonetheless, the role of lipid metabolism in microglial function remains an emerging field.

Microglia are involved in several homeostatic brain functions such as surveillance, phagocytosis, and maintenance of myelin dynamics [9,10,11]. For example, microglia facilitate primary myelination [12,13,14], via phagocytosis of apoptotic oligodendrocytes and myelin debris during early development [3]. Similarly, microglial “clearance” of myelin derived lipids is required for optimal remyelination following demyelination events [11,15]. As microglia age, they lose their ability to efflux cholesterol effectively and accumulate excessive amounts of cholesterol-rich myelin debris, which forms crystals and impairs phagocytic capacity, contributing to the progression of neurodegenerative disease (ND) [16]. LD accumulating microglia (LDAM) have phagocytosis deficits, produce excessive amounts of reactive oxygen species (ROS) and pro-inflammatory cytokines, and are increasingly implicated in the pathogenesis and progression of neuroinflammatory diseases [17,18,19]. Preventing LD accumulation may be beneficial in maintaining microglial homeostasis to mitigate ND. However, the mechanisms and metabolic perturbations that lead to excessive LD accumulation have yet to be fully elucidated.

The canonical role of Lipoprotein lipase (LPL) involves the provision of free fatty acids (FFA) to key metabolic tissues via the hydrolysis of triglyceride-rich lipoproteins. Although LPL is expressed in the central nervous system (CNS) [20,21,22], its function is less clear. Recent RNA-seq data suggest that LPL is predominantly expressed in the microglia of the human and mouse brain [23,24]. Moreover, microglial LPL expression has been implicated in brain development, damage, and disease [25,26,27]. In addition, several LPL polymorphisms have been linked to increased AD risk [28]. For example, patients with AD show reduced LPL abundance in the hippocampus, and reduced LPL enzymatic activity in their cerebrospinal fluid (CSF) [27]. In contrast, patients with the gain-of-function LPL polymorphism (S447X) have reduced very-low-density lipoprotein triglycerides (VLDL-TGs), higher high-density lipoproteins (HDL), and reduced amyloid load in the hippocampus [28]. In vitro and modeling studies suggest that the S447X mutation removes residues in the C-terminal tail of LPL, exposing a receptor-binding region that contributes to increased LPL-mediated endocytosis [28,29]. Overall there is a growing body of literature to support the notion that LPL plays a key role in microglial metabolism and function [7,11,26,30]. We have previously shown that LPL is a feature of phagocytic and “reparative” microglia, and that LPL may support remyelination by facilitating lipid uptake and promoting fatty acid oxidation (FAO) while maintaining an anti-inflammatory microglial phenotype [11].

Since LPL regulates microglial lipid metabolism, we reasoned that LPL may play a key role in LD formation and accumulation. Here, we characterize the role of LPL in LD accumulation, lipid composition, and transcriptional regulation of lipid metabolism in microglia. We use in vitro and ex vivo systems to show that LPL maintains microglial lipid metabolism, and that a loss of microglial-LPL is associated with increased LD and cholesterol load, and reduced peroxisome proliferator-activated receptor (PPAR) signaling. Moreover, we show that supplementation with PPAR agonists can reverse LD accumulation in microglia lacking LPL. Our findings galvanize the importance of LPL in microglial lipid metabolism and highlight a role in regulating overall microglial metabolism and function.

## 2. Materials and Methods

### 2.1. BV-2 Microglia Culture

BV-2 microglia [31] were cultured at 37 °C with 5% CO_2_ in a solution comprising Dulbecco’s modified Eagle’s medium (DMEM) with 10% fetal bovine serum (FBS) and 1% penicillin/streptomycin (P/S). The cells were grown to ~75–90% confluence for all experiments. LPL was knocked down (KD) in these cells using shRNA targeting, as previously described [11]. Both shRNA control (Control) and LPL KD BV-2 microglia were grown in the same culture conditions and for the same length of time for each experiment.

### 2.2. Primary Microglia Culture

All investigations using animals in this study were carried out using protocols (Ref #0115 and #0815) that were approved by the "ethical committee"; the University of Colorado Institutional Animal Care and Use Committee (IACUC), ensuring compliance with the recommendations in the Guide for the Care and Use of Laboratory Animals, Animal Welfare Act and PHS Policy.

CX3CR1^ERT^: LPL^flox/flox^ (Tamoxifen inducible, microglial specific LPL knock-down [MiLPLKD]) pups were sacrificed at post-natal day 0–2. Mouse brain tissues were collected and dissociated using 0.25% trypsin for 15–20 min at 37 °C in DMEM containing 10% FBS and triturated into single cells with a 10 mL pipette. The cells were plated at a density of 1 × 10^6^ cm^2^ on poly-d-lysine-coated (Corning, Glendale, USA) flasks. The medium was changed every alternate day using a 1:1 mix of fresh DMEM containing 10% FBS. Once the mixed glial cell culture reached 90% confluency (10–14 days), microglia were detached using trypsin (0.05%) in PBS and shaking. Microglia were collected and seeded onto poly-d-lysine coated coverslips. LPL KD was induced by tamoxifen treatment (40ng/mL for 3 days).

### 2.3. Neutral Lipid Staining in Primary Microglia and BV-2 Cells

After culture, cells were washed three times with HBSS. Primary microglia were fixed with 4% paraformaldehyde (PFA) for 5 min, while BV-2 cells were fixed with 4% PFA for 30 min. The fixed cells were washed three times with PBS. AdipoRED reagent (Lonza, 40× dilution) was used to stain the microglia for 10 min on the shaker, and then washed 3XPBS, each for 5 min. After washing, coverslips were mounted with DAPI and Fluoromount-G^TM^ (Invitrogen, Carlsbad, CA, USA).

### 2.4. Confocal Microscopy

Z-stack images were acquired on an Olympus laser scanning confocal microscope using a 100 × 1.4 numerical aperture oil-immersion objective with a 1.5× optical magnification. In BV-2 microglia imaging, Z-stack scans were taken at a 1.3 µm z dimensional resolution. In primary microglia imaging, Z-stack scans were taken at a 0.5 µm z dimensional resolution.

### 2.5. Lipid Droplet Quantification

Average LD volume and cell volume of microglia observed with confocal microscopy were quantified using ImageJ [32]. First, LD volume was determined by first applying a 32-bit filter and inverting each image. A left threshold of 30–40 was applied (range depending on variable background noise from stain). A watershed was applied to each image before the particles were analyzed for voxel volume. Next, total cell volume was determined by first applying a 32-bit filter to each image. A left threshold of 1 was applied, followed by a watershed filter to each image before the cells were analyzed for voxel volume. The voxel volumes were converted to µm by multiplying each dimension (x, y, and z) by the microscope resolution per pixel value of 0.13 µm (this value was verified using the image metadata analyzed by the voxel counter extension of ImageJ). Both LD volume and cell volume data from each image were taken as a sum across the z-stake for each image sample. Cells that were part within the z-stack images were given a fractional value based on their rough partial volume/the volume of an average cell within the series. Fused microglial cells were systemically counted as 1.5 cells within the analysis.

### 2.6. Lipidomic Analysis

Liquid chromatography (LC)/mass spectrometry (MSp) and normal phase hydrophobic interaction liquid chromatography (NP-HPLC)/MS were used to measure relative triglyceride (TG), diacylglycerol (DAG), cholesterol ester (CE), and phospholipid (PL) abundance. All lipids from BV-2 microglia (2 × 10^6^ cells per sample) were extracted according to the method presented by Bligh and Dyer [33]. For LC/MS analysis, the lipid extract was introduced onto the mass spectrometer and the lipid classes within the sample were separated using NP-HPLC with an Ascentis 5μ Si (15 cm × 2.1 mm) column (Supelco, Bellefonte, PA, USA). 30:40:7 hexane/2-propanol/water with a final concentration of 1 mM ammonium acetate (solvent A) and 30:40 hexane/2-propanol (solvent B) were used as a normal phase solvent system. In the initial mobile phase, solvent A was held at 46% for 3 min. Solvent A was brought from 46% to 70% over the span of 22 min, then from 70–100% over 5 min. Isocratic elution of 100% solvent A was carried out for 20 min. Within this gradient, TG, CE, and PLs were eluted at different time points based on the polarity of the lipids. The elution was electrosprayed directly into an interfacing triple quadrupole mass spectrometer (MDS Sciex API 3200; Applied Biosystems, Foster City, CA, USA). The newly formed positive and negative lipid-derived ions were detected by alternating polarity every 3 s and full scans of these ions were obtained from m/z 500–1100. The positive ions were electrosprayed using 4000 V with a declustering potential of 50 V, whereas the negative ions were electrosprayed using −4500 V with a declustering potential of −45 V. The mass spectrum across each NP-HPLC peak was assembled and the molecular ion abundance was determined and corrected for naturally occurring isotopes [34]. To determine the mole percentages of each lipid within the BV-2 microglia, negative [M − H]^−^, and positive [M + H]^+^ pseudomolecular analytes were used for negative and positive molecular species, respectively. The fatty acyl groups and glycerol backbones of each of the lipid species were determined by collision-induced dissociation (CID) of the [M − H]^−^ for negatively charged lipids. Additionally, the CID of the [M − CH_3_]^−^ analytes for positively charged lipids were analyzed in the negative ion mode at a high declustering potential (−150 V). The resultant demethylated positive anions were generated without adducts of acetate or chloride ions. 

### 2.7. Cholesterol Efflux Assay

BV-2 microglia were seeded in DMEM (10% FBS), at a density of 75 K per well of a 12 well plate. The cholesterol efflux assay was adapted from [35]. When the cells had adhered to the plate (around 1 h), [^3^H]cholesterol (Perkin Elmer) containing media was added to a final concentration of 0.5 μCi per well for 24 h. [^3^H]cholesterol-containing media was removed and the cells were washed three times in sterile PBS. Serum-free DMEM was added to the cells, with T0901317 (supplier) at 0–4 μMol/L for 20 h. Media was removed and 0.1 mL was added to 5 mL of Scintillation fluid and considered as “media counts”. The cells were washed three times with PBS and 0.5 mL of double-distilled water was added to the plates, which were then placed in a −80 °C freezer for one hour to help cells detach. After thawing, 0.1 mL of cell solution was added to 5 mL of scintillation fluid and the reads considered as "cell counts". Due to the slight delay in growth rate in the LPL KD cells, the counts were normalized for cell number and protein content. Cholesterol efflux was determined using the following equation (media counts/(media counts + cell counts) × 100) and expressed as a % of total.

### 2.8. Isolation of Microglia from Adult Mouse Brain

Adult mice were perfused with HBSS with calcium and magnesium, to prevent dissociation of LPL from the cell surface. Half of each adult mouse brain was minced with a sterile razor blade and placed in papain solution (Worthington, Lakewood, NJ, USA) #LK003150, prepared according to manufacturer’s instructions) and shaken at 100 rpm for 30 min at 37 °C. Tissue was triturated three times with a 10 mL, 5 mL, and 1 mL (pulled glass) pipette, respectively, filtered with a 70 μm cell strainer, and resuspended in Earle’s balanced salt solution with DNAse I. Dissociated cells were separated from myelin debris using the Worthington ovomucoid gradient and resuspended in PBS. CD11b microbeads (Miltenyi Biotech Inc, Auburn, CA, USA) were incubated with the cell suspension at 4 °C for 15 min. The cell suspension was added to a magnetic cell separation (MACs^®^) column (Miltenyi Biotech) and washed several times to remove non-CD11b^+^ cells. CD11b+ microglia were collected and immediately frozen in Trizol solution prior to RNA extraction.

### 2.9. RNA Isolation and RNAseq Library Preparation

The Agilent Tape Station 4200 RK6 screen tape was used to assess integrity of the Total RNA. 100 ng of Total RNA was used as input to construct mRNA libraries using the Nugen Universal Plus mRNA library prep kit catalog No. 0508. Library quality was assessed on a D1000 screen tape using the Agilent Tape Station 4200. Libraries were quantitated using the Qubit, diluted to 4 nM, and sequenced at a depth of 80 Million Paired End Reads 2 × 150 on the Illumina NovaSEQ6000 sequencer. bcl files were converted to FASTQ files using CASAVA 2.0 software. Data were analyzed using Basepair (https://www.basepairtech.com). Graphs of RNAseq data were generated using GraphPad Prism 6th Edition. Pathway analysis was completed using iDEP.91 software (http://bioinformatics.sdstate.edu/idep/). Pathways were analyzed in iDEP.91 using the fold change and corrected p-value data derived from Basepair analysis. Pathway enrichment was determined using gene set enrichment analysis (GSEA) and cap analysis of gene expression (CAGE), employing both GO functional categorizations and KEGG metabolic pathways to characterize enrichment.

### 2.10. Real-Time Quantitative Polymerase Chain Reaction (RT qPCR)

Total RNA was isolated from primary and BV-2 cells using RNeasy Plus Mini Kit (Qiagen, Valencia, CA, USA) according to the manufacturer’s protocol. Verso cDNA synthesis kit (Thermo Scientific, Waltham, MA, USA) was used to synthesize cDNA from RNA. Using housekeeping genes, GAPDH and UBC, to establish expression standards, gene expression levels relative to these standards were quantified by RT qPCR using SYBR Select Master Mix (Applied Biosystems, Foster City, CA, USA). The NIH sponsored NCBI primer-blast tool (https://www.ncbi.nlm.nih.gov/tools/primer-blast) was used to design primers (Supplementary Table S1) that crossed exon boundaries when possible. Using StepOnePlus instrument and software v2.3 (Applied Biosystems, Foster City, CA, USA), thermal cycling conditions for all RT qPCR studies were as follows: initial temperature of 50 °C for 2 min, 95 °C for 10 min, then 40 cycles of 95 °C for 15 s and 60 °C for 1 min.

### 2.11. Western Blot Analysis of Protein Expression

BV-2 cells were grown to 90% confluency and total protein was harvested using RIPA buffer (150 nM NaCl, 1% Triton X-100, 0.5% Sodium Deoxycholate, 0.1% SDS, 50 mM Tris pH 8.0, and Complete Protease Inhibitor Cocktail). Insoluble cell debris were removed by centrifugation, and protein containing supernatants were retained. Protein quantification was performed using the Peirce™ BCA protein Assay kit (Thermo). Total protein extracts were run on 12% SDS Separating Gel and transferred to nitrocellulose membrane. The membrane was blocked in PBST containing 5% non-fat milk and incubated with primary antibodies overnight (rabbit polyclonal anti-mouse LPL 1:1000, rabbit polyclonal anti-mouse PPARγ 1:500 (Proteintech, IL, USA) and mouse anti-GAPDH 1:1000 MAB374, (Millipore, Billerica, MA, USA)) overnight at 4 °C. Membranes were then incubated with secondary antibodies IRDye 800RD goat anti-rabbit, and IRDye 680RD donkey anti-mouse (Li-Cor, Nebraska, NE, USA) at 1:10,000 dilution. Protein bands were visualized using the Li-COR Odyssey with complementary software (Image studio).

### 2.12. PPAR Agonist Supplementation Experiments

BV-2 microglia were cultured in 1 mL wells for 24 h and washed prior to treatment with DMSO, 100 μM rosiglitazone, or 500 μM bezafibrate (Sigma, St. Louis, MO, USA) [36,37,38]. The cells were incubated with the treatments for 2 h at 37 °C with 5% CO_2_ and then washed. The wells were then prepared with neutral lipid, confocal microscopy, and lipid droplet quantification.

### 2.13. Statistical Analysis

For all analysis where two groups were compared, T-tests were performed and statistical significance stated where *p* < 0.05. When more than two groups were analyzed, a one way ANOVA (Analysis of Variance) was performed with Tukey post-hoc analysis, using an appropriately conservative correction for multiple comparisons.

## 3. Results

### 3.1. LPL Depletion Leads to Increased Lipid Droplet Accumulation

**Figure 1:**
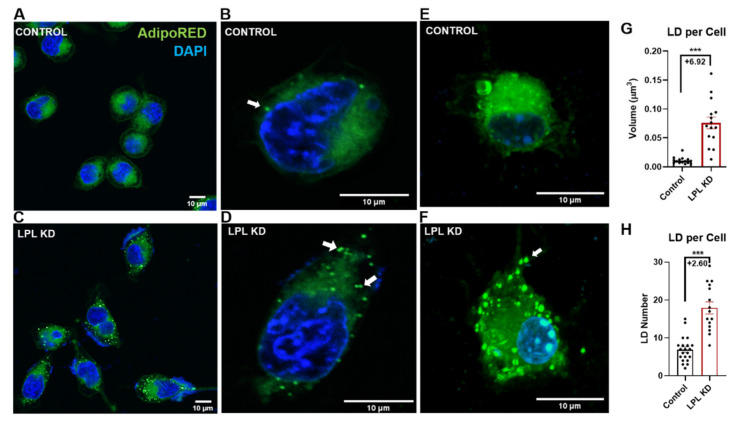
AdipoRed^TM^ staining of neutral lipid-containing lipid droplets in; (**A**,**B**). Control BV-2 microglial cells. (**C**,**D**). LPL KD BV-2 microglia. (**E**) Control primary microglia. (**F**). LPL KD primary microglia. White arrows indicate lipid droplets (LD). (**G**). Average number of LDs per cell in Control vs. LPL KD BV-2 microglia. N = 16 per group. (**H**). Average number of LDs per cell in Control vs. LPL KD primary microglia. N = 15 per group, * = *p* < 0.05, *** = *p* < 0.001.

Since microglial lipid and lipoprotein metabolism has increasingly been implicated in ND [39,40], and we have previously highlighted the importance of LPL in microglial lipid metabolism [11], we investigated the role of LPL in microglial LD accumulation. Neutral lipid accumulation in BV-2 (LPL KD vs. Control) and CX3CR1^ERT^: LPL^flox/flox^ primary microglia (LPL KD) was visualized using AdipoRED™. Comparative LD analysis revealed a marked increase in the LD accumulation and size of LPL KD and KD cells vs. control in both BV-2 and primary microglial cells (Figure 1A,F). BV-2 LPL KD cells contained +6.92-fold higher average total LD number per cell (*p* < 0.001 vs. control) (Figure 1G). In addition, LPL KD primary microglia had a +2.60-fold increase in the relative number of LDs per cell (*p* < 0.001 vs. control) (Figure 1H). These data strongly suggest that LPL deficiency within microglia is associated with increased lipid droplet accumulation. Since LD-containing microglia are dysfunctional [16,18], and distorted lipid profiles are known to alter the comportment of microglia [41], we performed lipidomic analysis to better understand the LPL-dependent changes in lipid composition.

### 3.2. LPL Deficient Microglia Show Marked Changes in Cholesterol and Phospholipid Metabolism

**Figure 2:**
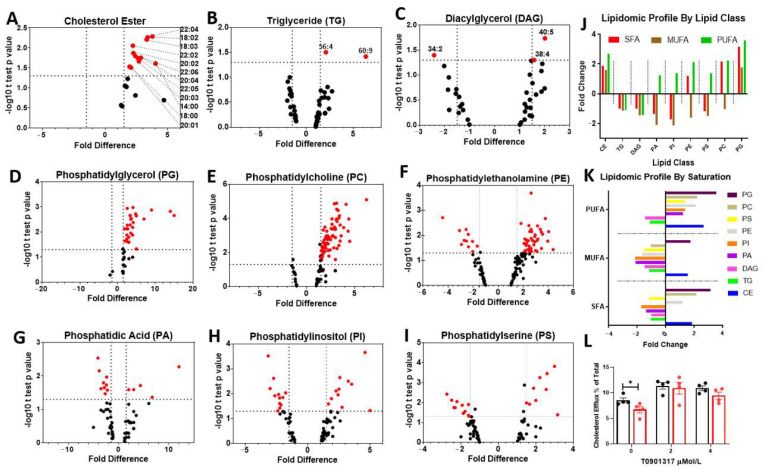
Relative lipidomics between Control and LPL KD (BV-2) microglia. (**A**–**I**). Volcano plots representing lipidomic profiles of CE, TG, DAG, PA, PG, PC, PE, PI, and PS in BV-2 microglia. Significance was determined as a ≥1.5 or ≤−1.5-fold difference in the relative abundance of lipid species in LPL KD vs. control with a *p*-value ≤ 0.05. (**J**)**.** Lipidomic profile by lipid class represents the difference in relative abundance of PUFA, MUFA, and SFA in control vs. LPL KD cells. (**K**). Lipidomic profile by saturation represents the degree of saturation in each lipid class in control vs. LPL KD cells. (**L**). Cholesterol efflux in control vs. LPL KD cells. where * = *p* < 0.05.

TG, DAG, CE, and PLs were extracted from BV-2 microglia followed by lipidomic analysis. BV-2 microglia were used for these assays due to the volume of cells needed (2 × 10^6^ per technical replicate) for all classes of lipids to be sufficienlty detected. The relative differences of each lipid class between LPL KD and control cells is shown (Figure 2). Overall, 236 unique lipid species were altered in LPL KD vs. control cells across all lipid classes (Figure 2A–C and Supplementary Table S2). In LPL KD, microglia CE was +2.61-fold higher than control BV-2 microglia (Figure 2J). As previously stated, increased intracellular cholesterol has been associated with inflammation and cellular dysfunction [16]. PLs were also altered in LPL KD cells. Phosphatidylglycerol (PG) is a mitochondrial PL that is increased in stressed or aged microglia and is converted to cardiolipin (CL) [42]. Interestingly, TG and DAG were mostly unchanged (Figure 2B,C). However, there was a significant increase in many PG species in LPL KD microglia (Figure 2D,J). Additionally, phosphatidylcholine (PC), which is known to alter the size and dynamics of LDs [43], and is a feature of reactive microglia [44], was increased in LPL KD cells (Figure 2E,J). Conversely, several lipid classes were reduced in LPL deficient cells compared to control cells. Certain species of phosphatidylethanolamine (PE) were reduced in the LPL KD cells, and appeared to cotnain mostly mono-unsaturated fatty acids (MUFAs) (Figure 2F,J). In addition, several species of phosphatidic acid (PA) (Figure 2G), phosphatidylinositol (PI) (Figure 2H), and phosphatidylserine (PS) (Figure 2I) were also reduced, particularly species containing saturated fatty acids (SFAs) and mono-unsaturated fatty acids (MUFAs) (Figure 2J,K).

Several studies have demonstrated the capacity of lipid supplementation to modify microglial phenotype and function [41]. Specifically, microglial supplementation with MUFAs has been shown to increase FA scavenging and incorporate polyunsaturated fatty acids (PUFAs) into TGs [41]. On the other hand, SFA supplementation incorporates more PUFAs into PLs, which are more readily peroxidized, contributing to downstream inflammation [41]. For these reasons, we analyzed the lipidomic profiles by saturation amongst the LPL KD vs. control cells. Interestingly, within the PL lipid class, both SFA (+1.26-fold) and PUFA (+1.92-fold) were more abundant in LPL KD microglia, while MUFA (−1.5-fold) was reduced when compared to control (derived from data in Figure 2K). Furthermore, MUFAs were decreased across all lipid classes except CE and PG (Figure 2J). Amongst the glycerolipids TG and DAG, LPL KD microglia had reduced PUFA (−1.30-fold), MUFA (−1.41-fold), and SFA (−1.01-fold), when compared to control (derived from data in Figure 2K). Taken together, these data suggest that LPL deficient microglia are associated with a lipidomic profile characterized by increased CE and reduced MUFA containing PLs, which is consistent with a pro-inflammatory phenotype. We hypothesized that reduced cholesterol efflux may explain the increase in LPL KD microglia. Efflux of [^3^H] cholesterol was measured at baseline and after supplementation with increasing concentrations of a pan-retinoic acid receptor (RXR) agonist T0901317. Interestingly we found that at baseline, cholesterol efflux was reduced in the LPL KD BV-2 cells compared to control, but this deficit could be reversed following RXR agonist supplementation (Figure 2L). 

### 3.3. Transcriptomic Analysis of LPL KD Microglia Highlights Impaired FFA Uptake, Reduced Cholesterol Efflux, and Inflammation

**Figure 3:**
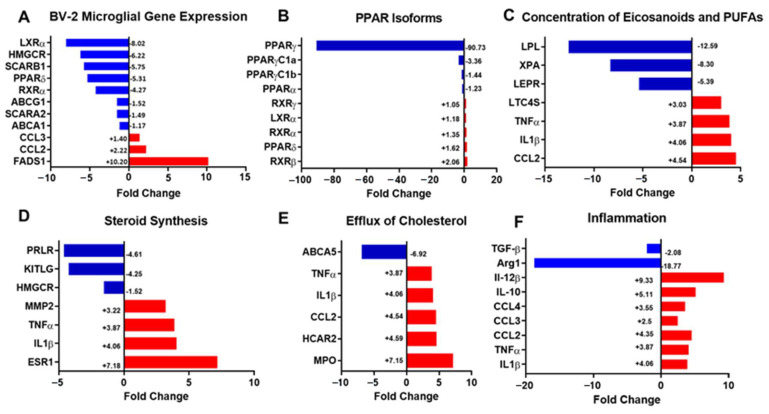
Altered expression of lipid metabolism and inflammatory genes in LPL KD BV-2 microglia, and microglia isolated from adult MiLPLKD mice. (**A**). Comparative gene expression between control and LPL KD BV-2 cells. B–F Comparative gene expression of CD11b^+^ microglia isolated from WT vs. MiLPLKD mice (**B**–**F**). Significance was determined as fold change ≥1.5 and *p* < 0.05.

To explore the mechanisms underlying the altered lipidomic profile of LPL KD cells, we measured the expression of genes involved in lipid metabolism in BV-2 control and LPL KD cells (Figure 3A, Supplementary Figure S2). Having previously shown that LPL deficient microglia have decreased phagocytosis of extracellular lipid [9], we first considered de novo cholesterol and FA synthesis as possible sources of increased LDs in the LPL KD cells. Interestingly, there was a trend towards reduced HMGCR expression, encoding the rate-limiting enzyme involved in de novo cholesterol synthesis (−6.22-fold, *p* = 0.07) (Figure 3A, Supplementary Figure S2C). FASN gene expression, encoding the rate-limiting enzyme involved in de novo FA synthesis, was not altered in the LPL KD vs. control cells (data not shown). Since long-chain PUFA (LC-PUFA) were increased across all PL classes (Figure 2K) we questioned whether these LC-PUFAs were undergoing modification for reutilization, so we examined elongase and desaturase expression. LPL KD microglia increased their expression of DEGS1 (+2.12-fold, *p* < 0.01), ELOVL1 (+2.57-fold, *p* < 0.05), ELOVL3 (+2.56-fold, *p* < 0.01), and ELOVL4 (+4.91-fold, *p* < 0.001) relative to control (Supplementary Figure S2E–H). Notably, expression of FADS1, a Δ5 desaturase that catalyzes the final step in the formation of both arachidonic acid (AA) and eicosapentaenoic acid (EPA), showed a marked +10.20-fold increase in the LPL KD cells (*p* < 0.05) (Figure 3A, Supplementary Figure S2D). AA, an omega-6 (n-6) PUFA, is a well-known precursor for pro-inflammatory eicosanoids [45], whereas EPA, an omega-3 (n-3) PUFA is a precursor for anti-inflammatory signaling molecules [46]. Whether FADS1 creates pro-inflammatory or anti-inflammatory precursor FAs, is dependent upon the FA being modified [47]. These data suggest that the lipidomic changes may serve to provide LPL KD microglia with secondary lipid mediators to promote inflammation.

Since master lipid gene regulators such as PPAR and liver X receptor (LXR) create heterodimers with RXR to regulate lipid metabolism and inflammation [48], expression of these regulators was examined. There was a -8.05-fold reduction in LXRα (*p* < 0.05) and a −4.27-fold reduction in RXRα (*p* < 0.05) in LPL KD vs. control cells (Figure 3A, Supplementary Figure S2K,I). Additionally, we observed a −5.75-fold, −1.17-fold, and −1.52-fold decreases in the downstream targets of the master lipid regulators: SCARB1 (*p* < 0.01), ABCA1 (*p* < 0.05), and ABCG1 (*p* < 0.01), respectively in LPL KD vs. control microglia (Figure 3A, Supplementary Figure S2L–O). We also observed a -5.31-fold reduction in PPARδ (*p* < vs. Control) (Figure 3A, Supplementary Figure S2J). Several studies utilizing BV-2 microglia have reported very low PPARγ expression [48,49]. Likewise, both our LPL KD and control microglia expressed PPARγ in low quantities.

While our transcriptomic findings from the analyses with BV-2 cells are consistent with lipidomics data, it is important to note that BV-2 cells are an immortalized microglial cell line, which are substantially different from primary cells. To extend our findings ex vivo, we performed transcriptomic analysis on CD11b+ microglia isolated from either MiLPLKD or WT mice. Ingenuity pathway analysis (IPA) was used to identify biological pathways that were altered in an LPL-dependant manner. The canonical ‘eicosanoid concentration’ (+1.98-fold, *p* < 0.001) and ‘PUFA concentration’ (+1.54-fold, *p* < 0.001) pathways were both increased in the MiLPLKD mice (Figure 3C), supporting our lipidomics data (Figure 2). Furthermore, both canonical pathways involving ‘steroid synthesis’ (−1.56-fold, *p* < 0.001) (Figure 3D) and ‘cholesterol efflux’ (−1.36-fold, *p* < 0.001) (Figure 3E) were decreased when compared to control. These data suggest that the loss of LPL is associated with reduced PPAR signaling, decreased cholesterol efflux, and increased concentration of lipid-derived secondary mediators. In addition, analysis of the transcriptome showed a marked increase in inflammatory markers and immune regulation (Figure 3F, and Supplementary Figure S2). Interestingly, anti-inflammatory markers were reduced in microglia isolated from MiLPLKD mice compared to WT mice (e.g., Arg1, TGF-β), consistent with our previous studies suggesting that LPL is a feature of alternatively activated microglia [11].

### 3.4. PPAR Activators Reduce LD Accumulation in Microglia

**Figure 4:**
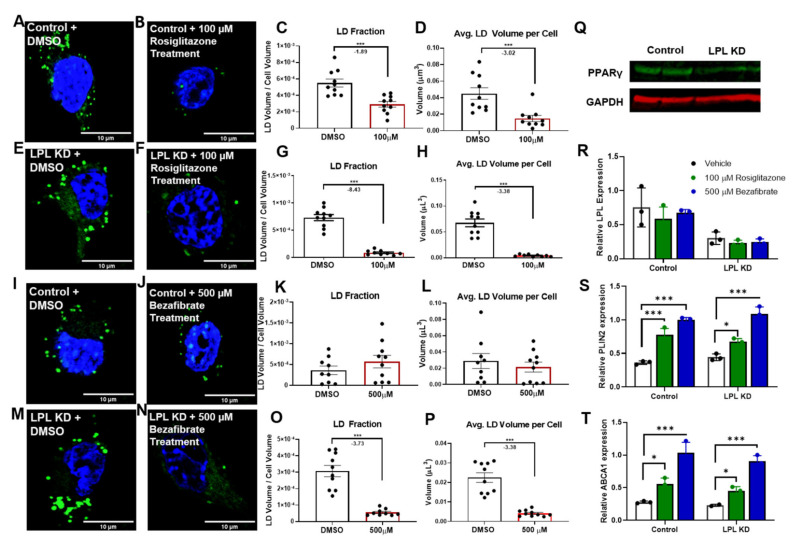
PPAR agonists rescue LPL KD microglia from LDAM phenotype. (**A**–**P**) Confocal microscopy of BV-2 control and LPL KD cells, treated with either vehicle (DMSO), 100 µM rosiglitazone, or 500 µM bezafibrate. Green dye indicates AdipoRed staining of neutral lipids. Blue DAPI staining indicates nuclei. Scale bar: 10 µm. (**A**–**D**). Quantitative analysis of relative LD fraction and volume in Control (BV-2) microglia treated with vehicle or 100 µM rosiglitazone. (**E**–**H**). Quantitative analysis of relative LD fraction and volume in LPL KD (BV-2) microglia treated with vehicle or 100 µM rosiglitazone. (**I**–**L**). Quantitative analysis of relative LD fraction and volume in Control (BV-2) microglia treated with vehicle or 500 µM bezafibrate. (**M**–**P**). Quantitative analysis of relative LD fraction and volume in LPL KD (BV-2) microglia treated with vehicle or 500 µM bezafibrate. *n* = 10 per group. (**Q**). Western blot showing PPARγ protein expression in 60 μg of total protein from either control or LPL KD cells. (**R–S**). Quantification of PPAR target genes in control vs. LPL KD BV-2 microglia treated with vehicle 100 µM rosiglitazone or 500 µM bezafibrate. * = *p* < 0.05, *** = *p* < 0.001.

PPAR agonists have been shown to protect against neuronal death and neuroinflammation, and also to promote CNS repair [50,51]. Since our data suggest that PPAR signaling is altered in LPL KD microglia, we supplemented BV-2 microglial cells with the PPAR agonists, rosiglitazone, and bezafibrate, in order to rescue LPL-dependant LD accumulation (Figure 4A–P). BV-2 cells were used in this experiment since the volume of cells required for multiple drug concentrations and time points could be easily generated. In addition to PPARγ gene expression being reduced in LPL KD cells, we also show reduced PPARγ protein expression in LPL KD microglia (Figure 4Q). It is well-known that rosiglitazone is a selective agonist of PPARγ, while bezafibrate is a pan-PPAR agonist [52]. Comparative LD analysis revealed a marked reduction in LD accumulation in LPL KD cells after rosiglitazone (Figure 4E–H) and bezafibrate (Figure 4M–P) treatment. Specifically, BV-2 LPL KD cells treated with rosiglitazone had a −8.43-fold lower total LD fraction of the total cell volume (*p* < 0.001) (Figure 4G) and a −3.38-fold reduction in the average total LD volume per cell (*p* < 0.001) (Figure 4H). Meanwhile, BV-2 LPL KD cells that were treated with bezafibrate showed a -3.73-fold reduction in the total LD fraction of the total cell volume (*p* < 0.001) (Figure 4O) and a −3.38-fold reduction in the average total LD volume per cell (*p* < 0.001) (Figure 4P). Similarly, BV-2 control cells treated with rosiglitazone showed a −1.89-fold reduction in the LD fraction of their total cell volume (*p* < 0.001) (Figure 4C) and a −3.02-fold reduction in the average total LD volume per cell (*p* < 0.001) (Figure 4D). Of note, when control cells were treated with bezafibrate the LD accumulation was unchanged (Figure 4K,L). To determine whether PPAR agonist supplementation enhanced downstream pathways, we measured the expression of PPAR target genes following rosiglitazone and bezafibrate treatment (Figure 4R–T). Interestingly, LPL was not increased following PPAR agonist supplementation (Figure 4R). In contrast, the PPAR target genes PLIN2 and ABCA1, were markedly increased following both bezafibrate and rosiglitazone supplementation (Figure 4S–T). Altogether, these data suggest that both bezafibrate and rosiglitazone rescue LPL KD microglia from increased LD accumulation. However, only rosiglitazone was able to reduce LD accumulation in control cells.

## 4. Discussion

Microglia play a key role in the onset and progression of NDs, such as MS and AD. In the search for potential disease-modifying targets, dysfunctional microglia subpopulations such as LDAM have recently been the subject of considerable investigation [16,18,19]. Generally, LDAM are characterized by excessive LD accumulation, defective phagocytosis, attenuated lipid efflux, and the excessive production of pro-inflammatory secondary messengers [16,18,19]. Understanding the mechanism(s) driving propagation of LDAM may help to identify potential therapeutic targets for NDs. We have previously shown that LPL KD microglia are polarized towards an inflammatory phenotype with impaired FAO and lipid uptake [11]. Here, we show that microglial LPL depletion is associated with the accumulation of intracellular LDs, a pro-inflammatory lipidomic profile, and a transcriptome that is associated with reduced cholesterol efflux and lipid processing. We also show that PPAR agonists can resolve microglial LD accumulation and could potentially provide therapeutic benefits in aging and NDs.

Here, we demonstrate that LPL deficient microglia have increased LD accumulation, characterized by increased LD volume, LD fraction, and number of LDs (Figure 1). Using both microglial cell lines and primary microglia, we show that the loss of LPL correlates with increased LD accumulation. Additionally, LDs within LPL KD microglia are morphologically different from those of control cells. Since LD morphology within microglia is an emerging research area with very few existing reports, key concepts involving mechanistic and physiologic relevance must be drawn from the extensive body of work involving adipocytes and hepatocytes. First, hepatocytes utilize adipose triglyceride lipase (ATGL) to breakdown large LDs, before utilization of lysosomal acid lipase (LAL) to further hydrolyze resulting small LDs [53]. Effectively, the presence of large LDs correlates with the impairment of LD-associated lipase activity [53,54]. Typically, LPL is bound to heparan sulfate proteoglycans (HSPG), to endothelium, or to the cell surface; however, LPL has been reported to exist in a "cryptic" inactive state within membrane-bound compartments of the cell [55]. Recent studies suggest that LPL is packaged into sphingomyelin-rich vesicles by the HSPG, Syndecan-1 (SDC1) [56], and exists as inactive filaments in adipocytes [57]. Whether LPL is processed in a similar fashion in microglia remains to be determined. Our lipidomic analysis supports our imaging data, as we demonstrate a higher overall molecular abundance of total lipids in LPL KD cells (Figure 2). Overall, more work is needed to determine the mechanism of altered LD composition and structure.

It is well established that microglial activation is facilitated by both intrinsic and extrinsic factors, leading to precise reprogramming of the microglial phenotype [58,59]. Altered lipid metabolism and extrinsic supplementation with various lipid substrates both have a major impact on microglial reprogramming [60,61,62,63,64]. For example, metabolic derangements of cholesterol lead to microglial activation, chronic inflammation, and progression of NDs [65,66]. Furthermore, impaired glial cholesterol efflux is associated with reduced cognition and worsening of neuroinflammation [16]. There have been conflicting reports regarding the cholesterol content within LDAM in brain aging and EAE models [16,18]. Studies determining LD composition in whole hippocampus and isolated microglia of young and aged mice report mainly glycerolipids with only a small proportion of cholesterol [18]. In contrast, Cantuti–Castelvetriet al. (2018) report a marked cholesterol content in phagocytes in an EAE demyelination model in aged mice [16]. In this study, we identify a marked increase in CE within the LPL KD LDAM (Figure 2). Since LD accumulation is a component of AD [67,68], and there are reduced levels of LPL in the dentate gyrus of patients with AD [27], it is plausible that reduced LPL in AD could contribute to LDAM physiology, increased CE load, microglial dysfunction, and disease progression.

These findings lead us to question whether the lipids accumulated within LPL KD microglia are created via de novo lipogenesis, from the redistribution of lipids, and resultant from decreased lipid efflux. LPS mediated-inflammation is known to promote LD accumulation in immune cells [19,69,70]. Chemokine (C-C motif) ligand 2 and 3 (CCL2 and CCL3), also known as macrophage inflammatory protein, are well-known chemotactic cytokines that promote inflammation. We demonstrated an increase in CCL2 in both BV-2 and primary microglia (Figure 3). CCL2 has been shown to influence LD biosynthesis [71] and pro-inflammatory polarization, and can interact with other inflammatory mediators [72]. Therefore, these data suggest elevated CCL2 expression in LPL KD microglia is associated with pro-inflammatory polarization and may contribute to LD accumulation.

Lipids within LDs are often reutilized in the production of immune mediators [73]. Additionally, certain metabolic states such as glucose starvation in microglia lead to lipid redistribution rather than de novo FA synthesis [74]. Our findings suggest that a potential source of LD accumulation is reduced cholesterol efflux capacity, along with a redistribution of lipid towards eicosanoids and PUFAs (Figure 2 and Figure 3). In support, transcriptome analysis of microglia isolated from MiLPLKD mice show an increase in pathways involving ’concentration of eicosanoids and PUFAs’, as well as a decrease in the ’cholesterol efflux’ pathway. This corroborates our lipidomic data, which shows increased CE and PUFAs in LPL KD microglia (Figure 2). Furthermore, ’steroid synthesis’ pathways are reduced (Figure 3), suggesting that increased CE accumulation in LPL KD microglia is associated with impaired cholesterol efflux rather than de novo lipogenesis or cholesterol synthesis. Additionally, FAS was unchanged; however, FADS1 was markedly increased in the LPL KD microglia (Figure 3A and Supplementary Figure 1D). These data, along with the reduction in FA scavenger proteins, suggest that FAs in the LPL KD microglia are not created via de novo FA synthesis, nor are they mainly derived from increased phagocytosis, but are rather redistributed from other saturation types to PUFAs, which serve as a source for the production of immune mediators, such as eicosanoids.

Impaired lipid efflux and lipid redistribution observed in LPL KD microglia may stem from reduced activity of nuclear lipid gene regulators. PPAR isomers are master regulators of hundreds of target genes involved in FAO, cholesterol and lipoprotein metabolism, LD formation, and inflammation [75,76]. FFAs act as PPAR agonists facilitating translocation to the nucleus and dimerization to RXR [77,78]. The PPAR-RXR dimer binds peroxisome proliferator response elements (PPRE) to activate the transcription of genes within the ATP-binding cassette (ABC) family and genes involved in FA scavenging and lipid metabolism [50,51,76,79]. Importantly, ABC proteins are integral to a diverse array of cellular processes, especially in the process of cholesterol efflux. Since LPL is well-known to hydrolyze TGs, and have potential phospholipase activity [80], LPL-derived FFAs may potentially serve as ligands for PPAR-RXR binding. In our BV-2 LPL KD LDAM, we demonstrate a reduction in master regulator genes: LXR, RXR, and PPARδ, along with their gene targets: ABCA1, ABCG1, SCARA2, and SCARB1 (Figure 3). Additionally, in primary microglia we demonstrate a dramatic reduction in PPARγ expression (Figure 3). With these data, it is plausible to deduce that a reduction in LPL is associated with reduced FFA uptake, which could otherwise act as natural agonists for PPAR and LXR, thus impeding cholesterol efflux capacity. It is important to note that the LXR-RXR heterodimer binds cholesterol breakdown products before translocating to the nucleus to bind a PPRE associated with the expression of many anti-inflammatory and cholesterol efflux promoting genes. Future studies examining the effects of LXR agonists on LD reduction in LDAM will provide a better understanding of LXR’s therapeutic potential.

PPAR agonists have often been associated with their ability to regulate CNS inflammation and demyelination [81,82]. In support of the idea that reduced PPAR signaling drives LDAM physiology, we have demonstrated that in vitro treatment with PPAR agonists rescue LPL KD BV-2 microglia from excessive LD accumulation (Figure 4). Interestingly, the PPARγ agonist, rosiglitazone, has a greater reduction of LD accumulation in LPL KD cells than a pan-PPAR agonist bezafibrate. The importance of selectively targeting PPARγ over other PPAR isoforms is further supported by rosiglitazone’s ability to further reduce LD accumulation in control cells when compared to the unchanged LD accumulation with bezafibrate (Figure 4). As previously stated, BV-2 microglial cells have been reported to have an absence of PPARγ expression [48,49], yet a drastic reduction in LD accumulation was observed following rosiglitazone supplementation. It is important to note that several reports have outlined the interplay between PPAR and the Janus kinase-STAT (Jak-STAT) system [36,82]. Park, et al. (2002) demonstrated that rosiglitazone and 15d-PGJ_2_ can act independently of PPARγ, to yield anti-inflammatory effects [36]. Although the effect of PPAR agonists on microglial LD accumulation is encouraging, LPL-independent effects may be at play in the PPAR agonists experiments, and therefore we must execute caution while interpreting these data. Further work is needed to define the mechanisms underlying the robust decrease in LD accumulation following PPAR agonist supplementation. 

A potential caveat of the present study is the use of immortalized BV-2 murine microglia. These cells are useful, particularly when genetically transformed, and when experimental endpoints require a large number of cells; these are yields that are often difficult to obtain by primary culture from either post-natal or adult mice. However, several reports have indicated that differences between immortalized cell lines and primary murine microglia are not insignificant [83,84]. In particular, while BV-2 and primary microglia respond similarly in migration and activation experiments, BV-2 cells show a damped cytokine release [83]. Since cytokine release is not the focus of these experiments, the use of BV-2 cells in this study remains valid. Nonetheless, it will be important to determine the differences in lipid and lipoprotein metabolism between BV-2 and primary microglia in future studies. In addition, it will be important to extend our studies regarding the role of LPL in microglia to in vivo models and eventually human IPSC-derived microglia.

We realize that the accumulation of LDs in microglia lacking LPL appears counter- intuitive, since LPL is largely involved in the uptake of lipids. Indeed, studies using rodents with a heart-specific LPL deficiency have shown that TG-containing LDs are reduced in the heart [85]. The differences in LD dynamics may relate to the complex and tissue-specific transcriptional and post-transcriptional regulation of LPL. In addition, there are major differences in the substrate availability of LPL in the heart compared to the brain. LPL in the heart has access to circulating TGs, which are absent from the brain. In the heart, LPL facilitates the uptake of FFAs into the cell, which are a preferred energy substrate. Therefore, it is expected that LPL deficiency would lead to reduced TG-accumulation in the heart where FFA and TG flux are in rapid turnover, but not the brain where glucose is the major substrate. The data presented in this manuscript, and our recently published studies, suggest that LPL is involved in the polarization of microglia. It is well-established that the pro-inflammatory polarization of the microglia results in LD accumulation. Therefore, our data suggest that LD accumulation in microglia lacking LPL is associated with inflammatory polarization rather than substrate utilization.

## 5. Conclusions

In summary, our findings highlight LPL as a key regulator of overall microglial lipid metabolism and a potential contributor towards LD accumulation. Our observations suggest that microglial LPL provides lipids/FFA to microglial to maintain FA and cholesterol metabolism, PPAR signaling, and cholesterol efflux. Thus, the presence of LPL prevents LD accumulation and polarization to a pro-inflammatory lipidomic profile to maintain microglial homeostasis. Moreover, PPAR agonists are not only able to rescue LPL KD cells from LD accumulation but also reduce LD accumulation in control cells, which may be beneficial in aging and in the treatment of NDs. We expect that the interaction between LPL, PPAR isomers, and LD accumulation will be further investigated to develop future therapies for aging and NDs.

## Data Availability

The data presented in this study are available in Supplementary Material here at www.mdpi.com/xxx/s1.

## Supplementary Materials

The following are available online at https://www.mdpi.com/2073-4409/10/2/198/s1, Table S1: Forward and reverse primer sequences by gene for qPCR, Table S2: Hydrocarbon chain profiles for PA, PC, PG, PI, PS, and PE, Figure S1: AdipoRED^TM^ staining of neutral lipid containing lipid droplets (LD)s in either Control or LPL KD primary microglia., Figure S2: Gene expression in BV-2 microglial LPL KD vs. control cells, Supplemental Figure 3: IDEP pathway analysis of relative gene expression differences in CD11b+ microglia isolated from 16 week old WT and MiLPLKD mice, Supplemental Figure 4: Western blot analysis showing reduced LPL in primary mouse LPL KD microglia.
