# Supplementary material for: Lipoprotein Lipase Regulates Microglial Lipid Droplet Accumulation
Source: Cells. 2021 Jan 20;10(2):198. doi: 10.3390/cells10020198 (PMC7909280; doi:10.3390/cells10020198)
Supplement: Supplementary file 1 [file cells-10-00198-s001.pdf]

Gene	Accession	Forward	Reverse
PPARy	NM_001308352.1	GACGCGGAAGAAGAGACCTG	GGAATGCGAGTGGTCTTCCA
PPARd	NM_011145.3	ATCGTCAACAAAGACGGGCT	GCTTCTACCTGGGGCACATT
RXRA	NM_011305.3	CCAAACATTTCCTGCCGCTC	AGGGTCATTTGGTGAGCTGG
NR1H3 (LXRA)	NM_013839.4	AGTTAGTCTGGTGGGGAAGC	CAACTCCGTTGCAGAATCAGG
ABCA1	NM_013454.3	TCCTTGGGGACAGAATTGCC	GCGTGTCACTTTCATGGTCG
ABCG1	NM_009593.2	CCTGCCTCCTCTTCTACCCT	TGCCTTGGGTTTGGGTTTCT
SCARB1	NM_016741.2	AGCCAAGCTATAGGGTCCTG	AGATCCGTCCTCTGTGGGAA
SCARA2	NM_010766.3	CGAATCTTTCCAACGCGTCC	TCTCTGTGCCCCGACAATTC
CCL2	NM_011333.3	CTGCTGTTCACAGTTGCCG	GCACAGACCTCTCTCTTGAGC
CCL3	NM_011337.2	TACAAGCAGCAGCGAGTACC	GAGCAAAGGCTGCTGGTTTC
HMG-CoAR	NM_008255.2	ACGATCCTTCCTTATTGGCGG	CACAGTCCTTGGATCCTCCG
ELOVL1	NC_000070	CCTGGTTTCCACTGAGTCCT	ACCCCATCAGAGGGTAGCTT
ELOVL2	NM_019423.2	GAAGGTGATGTCCGGGTAGC	CATGGACGCGTGGTGATAGA
ELOVL3	NM_007703.2	TACTTCTTTGGCTCTCGCCC	AGCTTACCCAGTACTCCTCCA
ELOVL4	NM_148941.2	CTGGAGGCCAAGCGTTTTTC	ATGGTCACGTGGAACTGGAC
ELOVL5	NM_134255.3	CAGCTTGCTTCTGTTCCCG	CTTTGACTCTTGTATCTCGGGG
ELOVL6	NM_130450.2	CACCCGAACTAGGTGACACG	CCGCAAGGCGTAGTAAGAGT
ELOVL7	NM_029001.5	GAGAACCGGAAGCCCTTTGA	CAGGTGTGCACCATCCTCAT
FADS1	NM_146094.2	CGCCAAACGCGCTACTTTAC	CCACAAAAGGATCCGTGGCA
FADS2	NM_019699.1	CAGCCCCTTGAGTATGGCAA	TAGTAGCTGATGGCCCAAGC
DEGS1	NM_007853.4	GCAGAGCGGGTCCACAG	TACTTTGCTAAGATCTCCTTGCG

Supplementary Table 1 | Forward and reverse primer sequences by gene for qPCR.

PA	14:0/16:0	14:0/18:1	16:0/18:1	16:1/18:1	18:0/20:1	18:0/20:2	18:0/22:4
	14:0/16:1	16:0/16:1	16:0/20:1	16:1e/18:1	18:1/18:1	18:0/20:3	18:0/22:5
PC	14:0/16:0	16:0/20:4	16:0/22:4	16:1/20:4	18:0/22:5	18:0/16:0	18:1/16:0
	14:0/18:0	16:0/22:4	16:0/22:5	16:1/22:4	18:0/22:6	18:0/18:0	18:1/18:0
	14:0/18:2	16:1/18:2	16:0/22:6	16:1/22:5	18:1/18:2	18:0/18:2	18:1/18:2
	16:0/16:0	16:0/14:0	16:1/14:0	16:1/22:6	18:1/18:3	18:0/20:1	18:1/20:2
	16:0/18:0	16:0/16:0	16:1/16:0	18:0/18:2	18:1/20:2	18:0/20:2	18:1/20:3
	16:0/18:2	16:0/18:2	16:1/18:0	18:0/20:1	18:1/20:3	18:0/20:3	18:1/20:4
	16:0/18:3	16:0/20:1	16:1/18:2	18:0/20:2	18:1/22:4	18:0/20:4	18:1/22:4
	16:0/20:1	16:0/20:2	16:1/20:1	18:0/20:3	18:2/18:2	18:0/22:4	18:1/22:6
	16:0/20:2	16:0/20:3	16:1/20:2	18:0/20:4	18:2/20:1	18:0/22:5	20:1/20:4
	16:0/20:3	16:0/20:4	16:1/20:3	18:0/22:4	18:0/14:0	18:0/22:6	20:1/22:5
PG	14:0/18:2	16:0/20:4	18:0/20:1	18:0/22:6	18:1/20:4	18:2/20:2	18:0/18:1
	16:0/18:2	16:1/18:2	18:0/20:2	18:1/18:2	18:1/22:5	18:2/20:3	18:1/16:0
	16:0/20:1	18:0/18:0	18:0/20:3	18:1/20:2	18:2/18:2	18:2/20:4	18:1/18:0
	16:0/20:2	18:0/18:1	18:0/20:4	18:1/20:3	18:2/20:1	18:0/16:0	18:1/18:2
	16:0/20:3	18:0/18:2	18:0/22:5				
PI	14:0/18:2	16:0/18:2	16:1/18:0	18:0/18:2	18:0/18:1	18:0/20:1	18:1/20:1
	14:0/20:4	16:0/22:4	16:1/18:2	16:1/18:1	18:1/18:2	18:0/20:2	18:1/16:0
	14:0/22:5	16:0/18:1	16:0/18:1	16:1/18:2	18:0/18:0	18:1/18:1	18:1/18:1
	14:0/22:6	16:0/16:0					
PS	14:0/16:0	16:0/18:1	16:1/18:1	18:0/20:2	18:0/18:2	18:0/22:4	18:1/20:1
	14:0/18:1	16:1/16:1	16:1/20:1	18:0/20:3	18:0/20:3	18:1/20:4	18:1/18:1
	16:0/16:1	16:1/18:0	18:0/18:2	18:0/22:4	18:0/20:4	18:1/18:1	
PE	14:0/18:2	16:0/22:6	16:1/14:0	16:1/22:6	18:0/22:6	18:1/18:3	18:1/22:6
	14:0/20:4	16:0/16:1	16:1/16:1	16:1/22:6	18:0/20:4	18:1/20:4	18:2/20:3
	14:0/22:5	16:0/14:0	16:1/18:0	18:0/18:2	18:0/22:4	18:1/22:4	18:2/20:4
	16:0/18:2	16:0/16:1	16:1/18:1	18:0/18:3	18:0/22:5	18:1/22:6	18:2/22:4
	16:0/18:3	16:0/22:4	16:1/20:1	18:0/20:3	18:0/22:6	18:1/20:2	18:2/22:5
	16:0/20:3	16:0/22:5	16:1/20:3	18:0/20:4	18:1/20:4	18:1/20:3	18:2/22:6
	16:0/20:4	16:0/22:6	16:1/20:4	18:0/20:5	18:1/22:4	18:1/20:4	20:1/20:4
	16:0/22:4	16:1/16:1	16:1/22:4	18:0/22:4	18:1/22:6	18:1/22:4	20:1/22:5
	16:0/22:5	16:1/18:1	16:1/22:5	18:0/22:5	18:1/16:1		

Supplementary Table 2 | Hydrocarbon chain profiles for PA, PC, PG, PI, PS, and PE. Red text represents an increased molecular abundance of the respective hydrocarbon chain profiles, while blue text represents a relative decrease molecular abundance the respective profiles between LPL KD vs control cells. Profiles that are listed more than one time represent fatty acyl groups that originate from a different position on the phospholipid backbone (e.g. sn1, sn2, sn3).

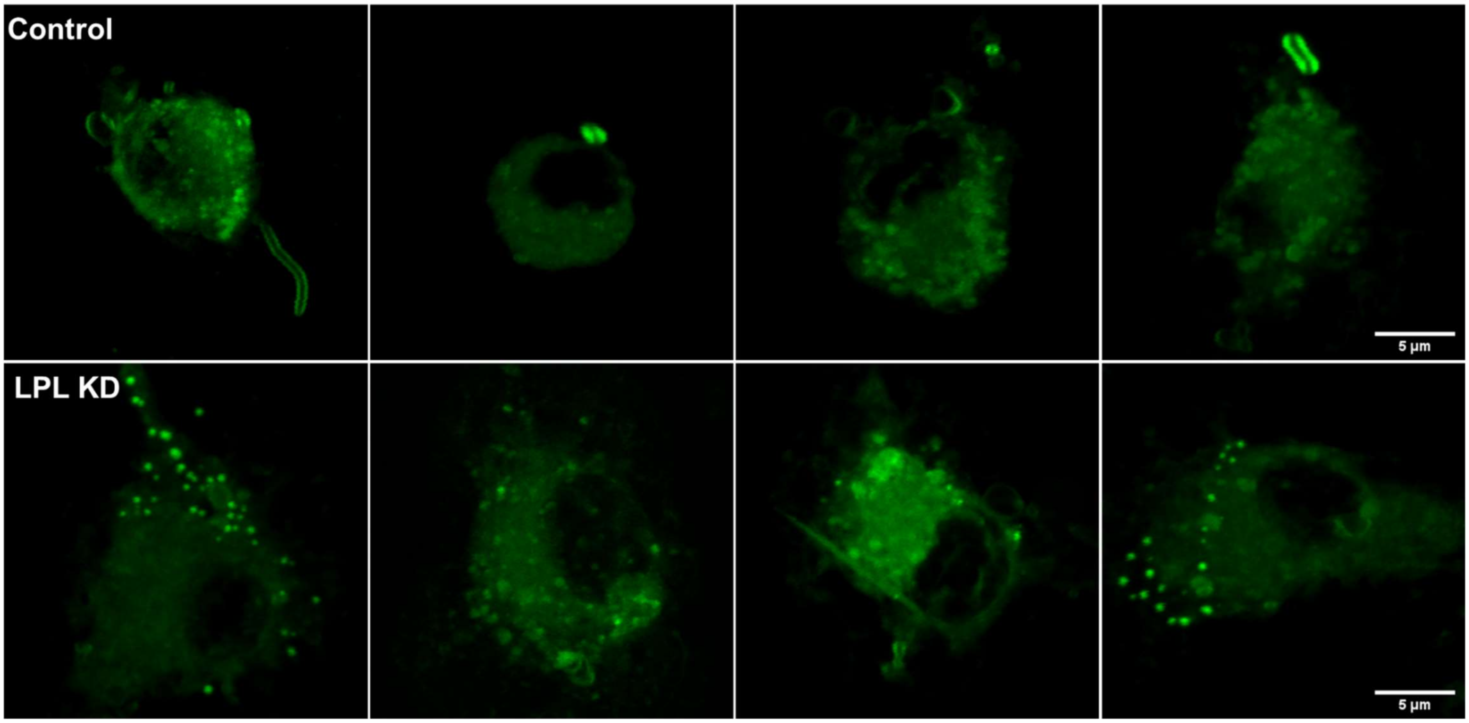

Supplementary Figure 1 | AdipoRED^{TM} staining of neutral lipid containing lipid droplets (LD)s in either Control or LPL KD primary microglia**.** (N=4 per group).

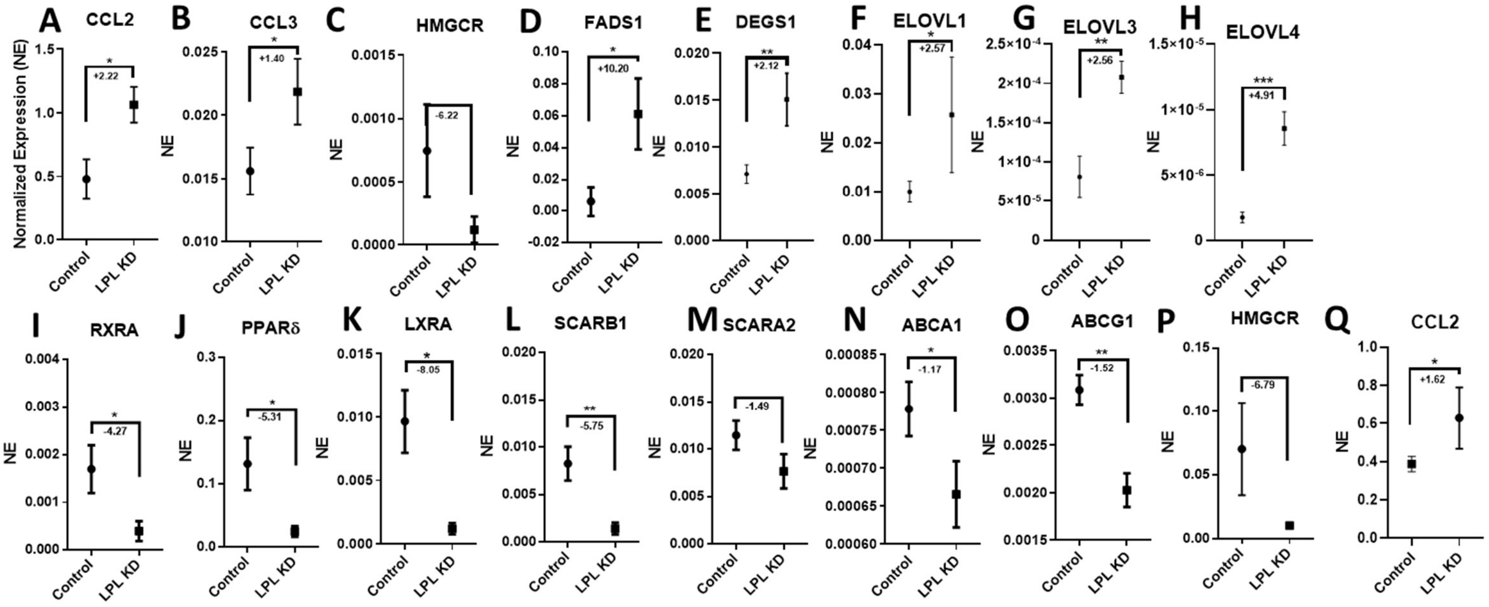

Supplemental Figure 2 | (A-O) Gene expression in BV-2 microglial LPL KD vs control cells. (N=3 per group). **(P-Q)** Gene expression in primary microglial LPL KD vs control cells. (N=3 per group). *P < 0.05. **P < 0.01. ***P < 0.001.

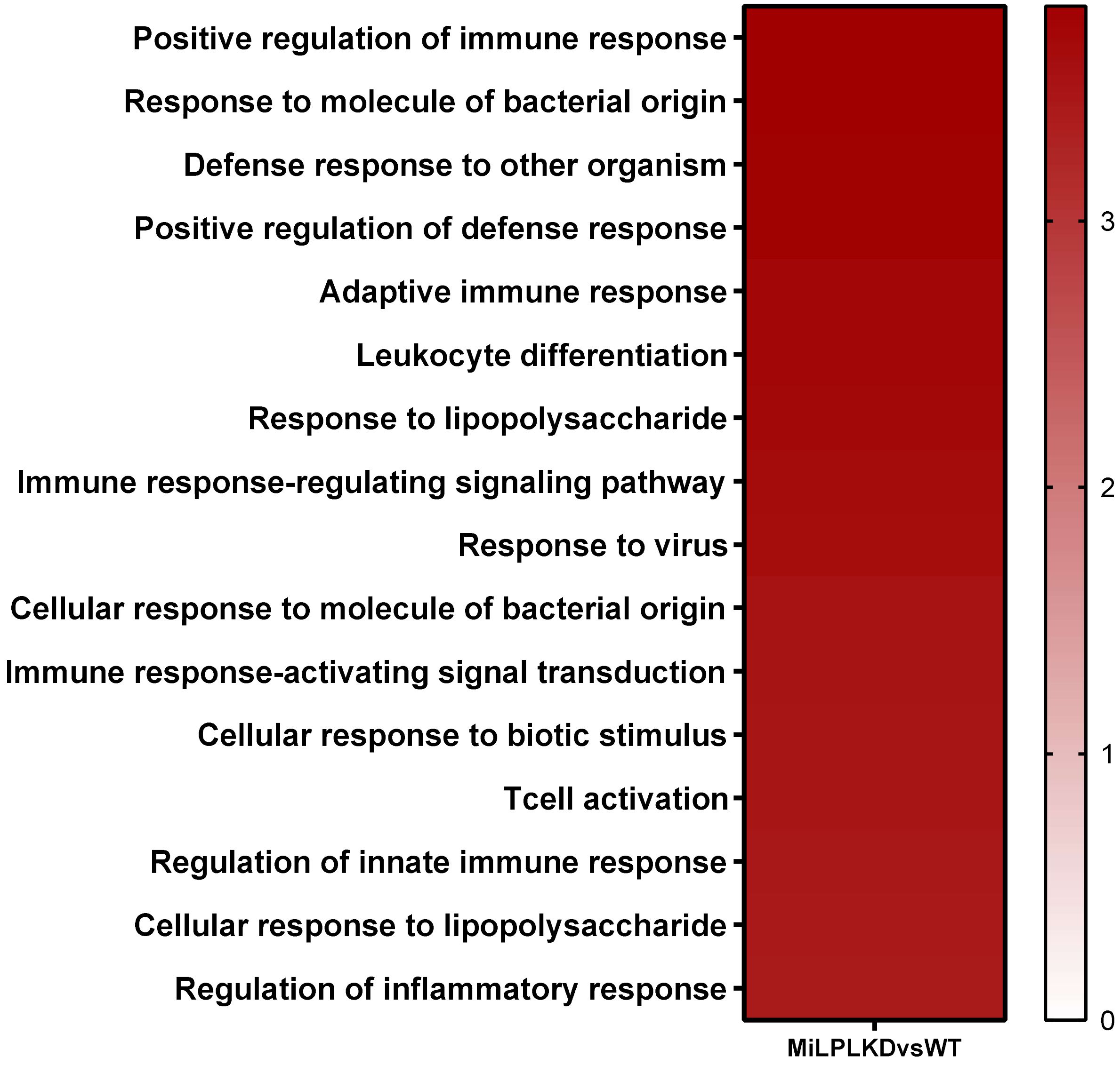

Supplemental Figure 3. IDEP pathway analysis of relative gene expression differences in CD11b+ microglia isolated from 16 week old WT and MiLPLKD mice.

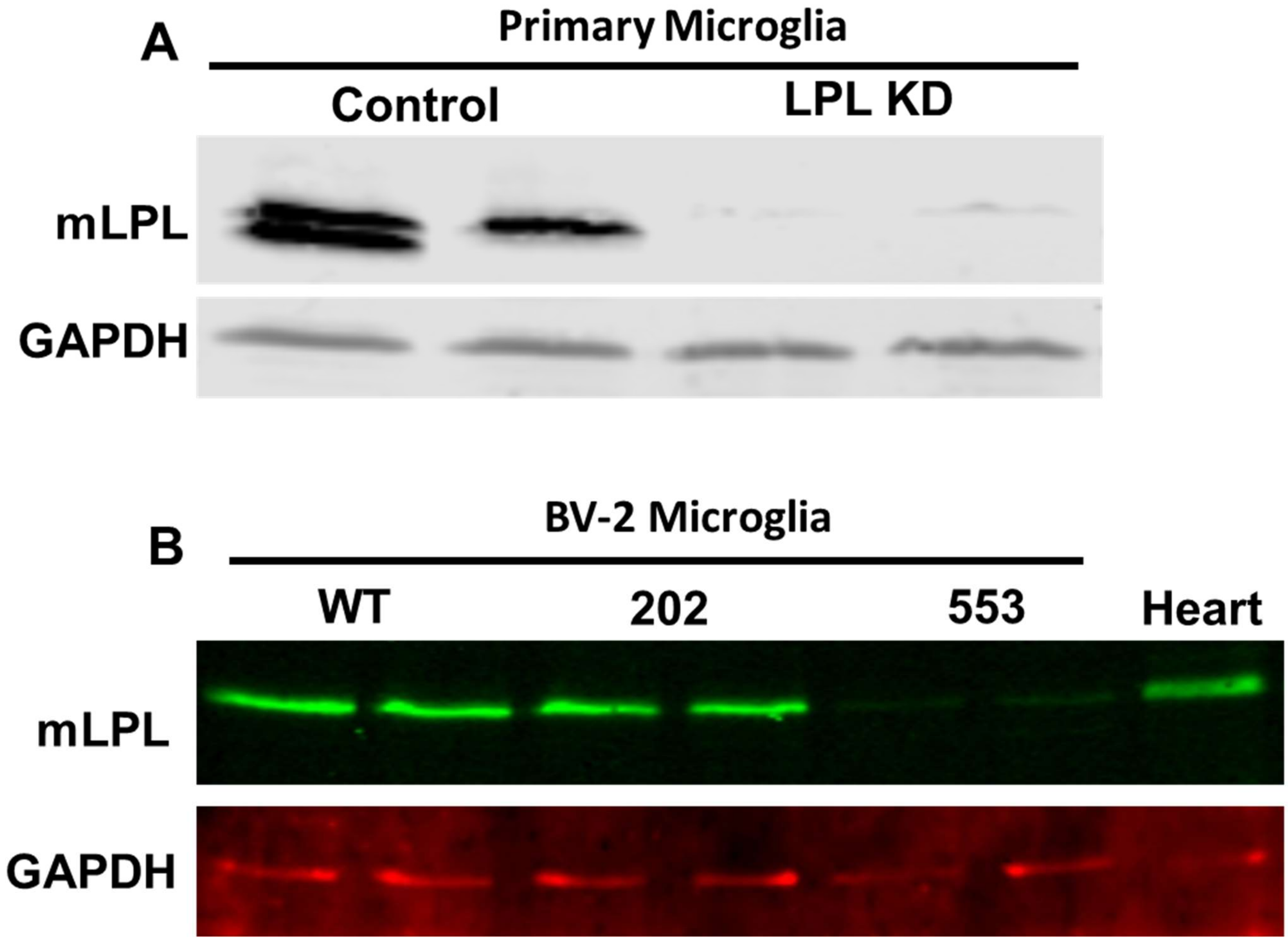

Supplemental Figure 4. Western blot analysis showing reduced LPL in primary mouse LPL KD microglia (**A**), and WT, 202 (shRNA control) and 553 (LPL KD) BV-2 microglia (previously shown in Bruce et al., 2018).
